# Supplementary material for: The Degradation of Deoxynivalenol by Using Electrochemical Oxidation with Graphite Electrodes and the Toxicity Assessment of Degradation Products
Source: Toxins (Basel). 2019 Aug 19;11(8):478. doi: 10.3390/toxins11080478 (PMC6723037; doi:10.3390/toxins11080478)
Supplement: Supplementary file 1 [file toxins-11-00478-s001.pdf]

Supplementary Materials: The Degradation of Deoxynivalenol by using Electrochemical Oxidation with Graphite Electrodes and the Toxicity Assessment of Degradation Products

Suli Xiong, Xiao Li, Changsong Zhao, Jingqi Gao, Wenjuan Yuan and Jie Zhang

Several common experimental materials were selected to treat DON, including zeolite, activated carbon (AC), activated carbon fiber (ACF), titanium (Ti) mesh, and graphite. Different mechanisms were considered when testing with these materials, and the detailed mechanisms of these tries were shown in Table S1. To ensure a consistent surface area, the surface area of these materials were measured with a surface area analyzer (GeminiVII 2390t, Micromeritics, USA) using the BET model. The ECO experiment was carried out u at the potential of 0.5 V for 30 min. All experiments were carried out at room temperature as well as magnetic stirring was performed at a speed of 1000 r/min to ensure samples were mixed completely.

Table S1. The materials and mechanism used to treat DON.

Number	Material	Mechanism	BET/$m^2\,g^{-1}$
T1	Zeolite	adsorption	623.1
T2	AC	adsorption	1243.1
T3	ACF	adsorption	1588.1
T4	Graphite	adsorption	421.0
T5	Ti mesh	ECO	293.4
T6	ACF	ECO	1588.1
T7	Graphite	ECO	421.0

Figure S1 shown the removal rate of DON in different treatment groups. All materials showed poor adsorbability towards DON. The adsorption rates of Zeolite, AC, ACF, and Graphite for DON were 3.96, 4.98, 4.88, and 6.96%, respectively. This was similar to some of the previous results [1], but the adsorption effect of AC on DON was quite different, which may be caused by the difference of experimental materials and experimental conditions. Interestingly, the results of ECO were quite different. The removal rates of DON by Ti mesh and ACF were 8.64% and 11.05%, respectively. However, the removal rate of DON by graphite electrode can reached 86.72%. In order to investigate the excellent DON removal rate of graphite electrode, we conducted subsequent comparison experiments between Ti mesh electrodes and graphite electrodes.

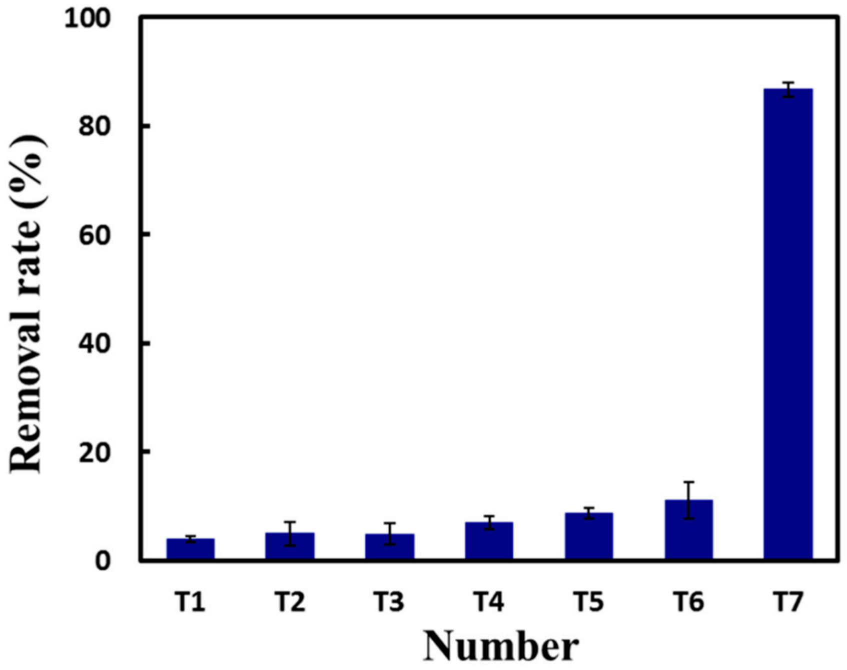

Figure S1: The removal rate of DON in different treatment groups. The values were expressed as means ± standard deviation (SD) (n = 3).

1. Avantaggiato, G.; Havenaar, R.; Visconti, A. Evaluation of the intestinal absorption of deoxynivalenol and nivalenol by an in vitro gastrointestinal model, and the binding efficacy of activated carbon and other adsorbent materials. *Food Chem Toxicol* **2004**, *42*, 817–824, doi:10.1016/j.fct.2004.01.004.
